# Glycyrrhiza uralensis Fisch. suppresses cell migration via ROS and JAK/STAT signalling pathways in *Drosophila*

**DOI:** 10.3389/fphar.2025.1549920

**Published:** 2025-06-27

**Authors:** Fangfei Zhou, Qingge Lu, Lingyu Kong, Sitong Wang, Haixia Zhang, Meng Zhao, Yue Hu, Fanwu Wu, Chenxi Wu

**Affiliations:** ^1^ Hebei Key Laboratory of Integrated Traditional Chinese and Western Medicine for Diabetes and Its Complications, College of Traditional Chinese Medicine, North China University of Science and Technology, Tangshan, China; ^2^ Department of Dermatology, Xiong’an Xuanwu Hospital, Xiong’an, China; ^3^ Department of Proctology, Tangshan Hospital of Traditional Chinese Medicine, Tangshan, China; ^4^ North China University of Science and Technology Affiliated Hospital, Oncology of Chinese and Western Medicine, Tangshan, China

**Keywords:** *Glycyrrhiza uralensis* Fisch., cell migration, ROS, JAK/STAT, *Drosophila*

## Abstract

**Background:**

Cancer is a global public health crisis and the leading cause of death among middle-aged and older individuals, with its incidence increasingly shifting toward younger populations. Approximately 90% of the patients succumb to advanced metastasis, and effective treatments remain elusive. The specific molecular mechanisms underlying cancer cell invasion and migration remain poorly understood, hindering the development of effective targeted therapies. Therefore, inhibiting or reversing cancer cell invasion and migration may be crucial for reducing mortality. Our previous research revealed that the five drugs (FD), derived from Xuefu Zhuyu Decoction (XFZYD), play a significant role in inhibiting cell migration.

**Hypothesis/purpose:**

This study aims to explore the main drug components of FD and investigate the underlying mechanism in inhibiting cell migration.

**Methods:**

We used the *Dro*s*ophila ptc>scrib-IR* cell migration model to investigate the effects of FD. FD was disassembled and analyzed using an orthogonal design. Drug extracts were prepared and administered to *Drosophila* larvae. We assessed the effects of FD on cell migration, reactive oxygen species (ROS) levels, and gene expression.

**Results:**

In FD disassembled recipes and orthogonal test design, a significant difference was observed in the intervention with or without *Glycyrrhiza uralensis* Fisch. (GUF) in migrating cell number (*P* < 0.01), which emerged as a more potent inhibitor of FD from XFZYD in cell migration. High-performance liquid chromatography revealed that GUF and its extract contained effective medicinal components, namely glycyrrhizic acid, liquiritin, liquiritigenin, and glycyrrhetinic acid. Moreover, GUF at 4.0 mg/mL displayed strong inhibitory effect in migrating cell number and distance when compared with model, XFZYD or FD. Excessive ROS can activate the JAK/STAT signaling pathway and promote the EMT process. GUF inhibited *ptc*>*scrib-IR-*induced cell migration by reducing ROS levels, JAK/STAT signalling, and the transcription of *upd2*, *upd3*, *hop* and *socs36E*. Finally, GUF rescued the altered expressions of the epithelial-mesenchymal transition (EMT)-related proteins, including matrix metalloproteinase 1 (MMP1), β-integrin and E-cadherin, triggered by cell migration.

**Conclusion:**

Our findings demonstrate that GUF may serve as a promising candidate for targeting advanced metastatic tumors by suppressing ROS-mediated JAK/STAT signaling and EMT.

## Graphical Abstract

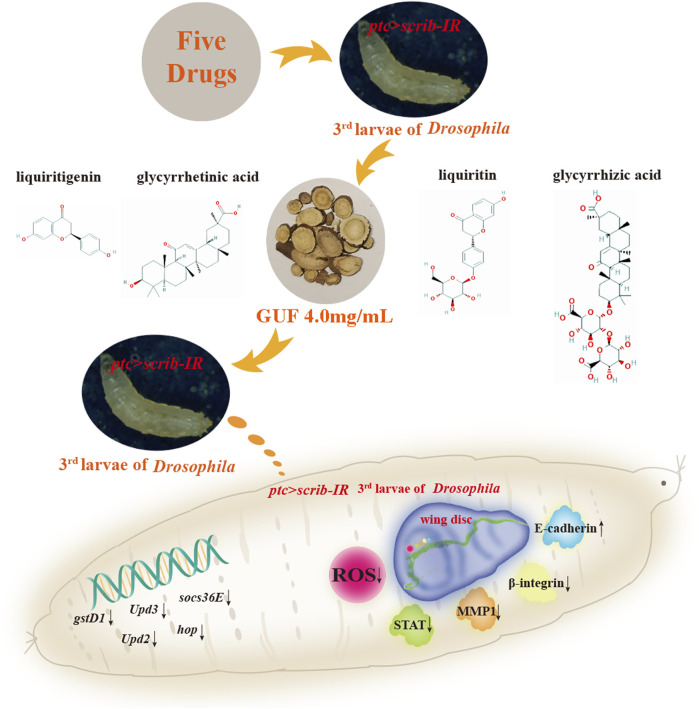

## 1 Introduction

More than 90% of patients with malignant tumours succumb to advanced metastases (Jonckheere et al., 2022). Recent evidence indicates a shift in cancer incidence from older adults to middle-aged. individuals (Siegel et al., 2024). Although early screening, diagnosis, and treatment have significantly lowered cancer mortality rates, treatment options for late-stage metastasis remains limited (Shi et al., 2024). This underscores the urgent need to identify novel, effective, and sustainable natural compounds to target metastasis and inhibit cell migration. Epithelial-mesenchymal transition (EMT), a reversible process, plays a crucial role in this progression by driving epithelial cells to gradually lose polarity and intercellular adhesion, while enhancing motility and promoting a mesenchymal phenotype, ultimately facilitating invasion and metastasis (Huang et al., 2022).

Natural herbal medicines have been used to treat malignant tumours in China and globally for thousands of years, offering efficiency, low toxicity, and good tolerance (Wei et al., 2022). Among these is the traditional Chinese medicine (TCM) Xuefu Zhuyu Decoction (XFZYD), containing 11 herbs, including *Prunus persica* (L.) Batsch (Tao Ren), *Carthamus tinctorius* L. (Hong Hua), *Ligusticum chuanxiong* Hort. (Chuan Xiong), *Angelica sinensis* (Oliv.) Diels. (Dang Gui), *Paeonia lactiflora* Pall. (Chi Shao), and *Rehmannia glutinosa* Libosch. (Sheng Dihuang), *Bupleurum chinense* DC*.* (Bei Chaihu), *Citrus aurantium* L. (Fuchao Zhiqiao), *Glycyrrhiza uralensis* Fisch. (GUF) (Gan Cao), *Cyathula officinalis* Kuan (Chuan Niuxi), and *Platycodon grandiflorum* (Jacq.) A. DC. (Jie Geng). XFZYD has been shown to reduce the risk of colorectal cancer (Jhang et al., 2020) by modulating Notch, JNK, and caspase signalling (Wang S. et al., 2020; Wang et al., 2022).

Our previously study demonstrated that XFZYD regulates tumour and cell migration in a dose-dependent manner, with different concentrations producing varying, even oppossing effects. The optimal inhibitory concentration was identified as 12.5 mg/mL (Wang S. et al., 2020). Building on this dosage, we disassembled XFZYD and performed factorial design assays (Ye, 2022). Through this approach, we identified five natural herbs, including *C. aurantium* L. (Fuchao Zhiqiao) and *P. grandiflorum* (Jacq.) A. DC. (Jie Geng) and *B. chinense* DC. (Bei Chaihu), *C. officinalis* Kuan (Chuan Niuxi) and *G. uralensis* Fisch. (GUF, Gan Cao) (collectively called five drugs, FD), which played a central role in inhibiting migration. The optimal inhibitory effect was achieved at a concentration of 10.0 mg/mL (Ye, 2022). However, the specific active components and underlying molecular mechanisms of FD remain unclear, and warrant further investigation.

*Drosophila melanogaster* shares 75% of its pathogenic genome with humans (Halim et al., 2020) and offers genetic tools for studying human cancer phenotypes (Mirzoyan et al., 2019). About 90% of human cancers are epithelial; *Drosophila* larval wing discs, which resemble mammalian epithelial cells in morphology and biochemical function, effectively model the development of epithelium-derived cancers (Hanahan and Weinberg, 2000; Mirzoyan et al., 2019). *Drosophila* genes are easily manipulated, enabling the establishment of various stable tumor models through genetic techniques. For instance, activating Ras^V12^ expression through the FLP-FRT-mediated cloning technique induced benign tumor formation. When combined with the UAS/GAL4 dual-expression system to simultaneously mutate the tumor suppressor gene *scrib*, this approach generates a stable tumor infiltration and metastasis model (Elliott and Brand, 2008; Miles et al., 2011). In another model, the UAS/GAL4 dual-expression system was used to generate a stable *ptc*>*scrib-IR* line, in which *scrib* is specifically downregulated along the anterior/posterior (A/P) axis of the wing imaginal disc of *Drosophila* larvae (Elsum et al., 2012). This suppression induces EMT-like changes in the cells, and promotes extensive posterior invasion and migration (Elsum et al., 2012).

In this study, *Drosophila* was used as a model organism to establish the *ptc*>*scrib-IR* cell migration model, enabling investigation of the effects of disassembled FD components on cell migration and their regulatory mechanisms *in vivo*, with the aim of providing new insights and theoretical guidance for preventing and treating malignant tumours.

## 2 Materials and methods

### 2.1 Herb information

Supplementary Table S1 Wang S. et al. (2020); Wang et al. (2022) lists the 11 traditional Chinese herbs in XFZYD, including their Latin/Chinese names, batch numbers, origin, and dosages. The herbs were purchased from Hebei Linyi Tang Pharmaceutical Co., Ltd., China and identified by Professor Fanwu Wu at the College of Traditional Chinese Medicine, North China University of Science and Technology.

### 2.2 Disassembled recipes and orthogonal design of FD

**FIGURE 1:**
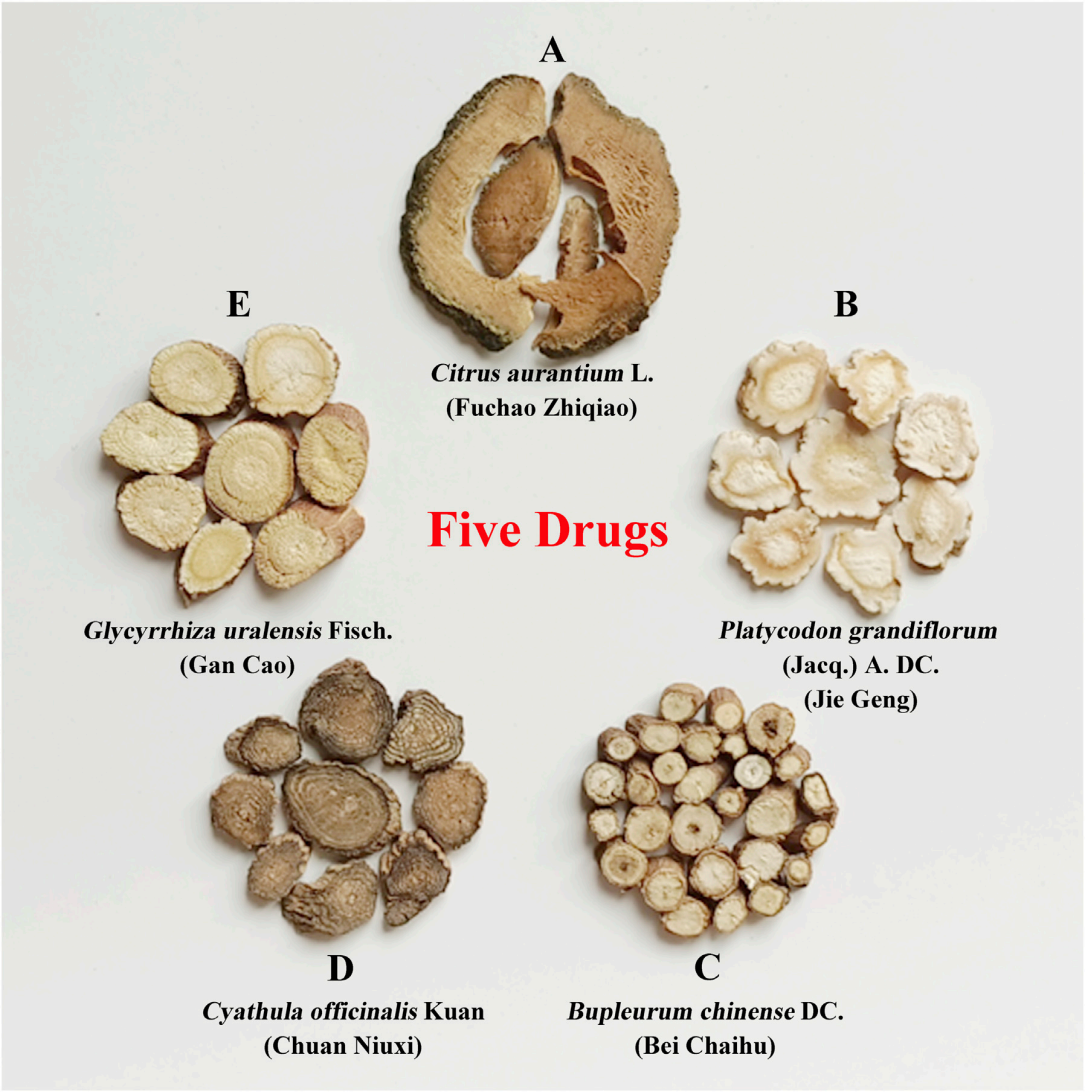
Disassembled recipes of FD. The five natural herbs in FD are divided into five influencing factors: **(A–E)**. **(A)** *Citrus aurantium* L. (Fuchao Zhiqiao); **(B)** *Platycodon grandiflorum* (Jacq.) **(A)**. DC. (Jie Geng); **(C)** *Bupleurum chinense* DC. (Bei Chaihu); **(D)** *Cyathula officinalis* Kuan (Chuan Niuxi); **(E)** *Glycyrrhiza uralensis* Fisch. (Gan Cao). FD: Five drugs.

FD comprises five natural herbs, each considered an independent factor: A, B, C, D, and E (Figure 1). Experimental groups were designed using an L8 (2^7^) orthogonal design table (Table 1), incorporating five main influencing factors (A, B, C, D, and E) and one interaction effect (A × B, representing two medicinal materials with opposing effects), each tested at two levels (level 1, drug treatment; level 2, no drug treatment). The drug concentrations for the eight experimental groups were calculated based on the FD molecular weight composition ratio at a total concentration of 10.0 mg/mL (Supplementary Table S2). Statistical analyses were performed using orthogonal analysis and one-way ANOVA.

**TABLE 1:** Orthogonal design using the five drugs disassembled recipes.

Experiment number	A	B	AxB	C	D	E	Blank	Group
1	1	1	1	1	1	1	1	A + B + C + D + E
2	1	1	1	2	2	2	2	A + B
3	1	2	2	1	1	2	2	A + C + D
4	1	2	2	2	2	1	1	A + E
5	2	1	2	1	2	1	2	B + C + E
6	2	1	2	2	1	2	1	B + D
7	2	2	1	1	2	2	1	C
8	2	2	1	2	1	1	2	D + E

A: *Citrus aurantium* L. (Fuchao Zhiqiao); B: *Platycodon grandiflorum* (Jacq.) A. DC. (Jie Geng); C: *Bupleurum chinense* DC. (Bei Chaihu); D: *Cyathula officinalis* Kuan (Chuan Niuxi); E: *Glycyrrhiza uralensis* Fisch. (Gan Cao). 1 (level 1): represent drug treatment; 2 (level 2): represent no drug treatment. The interaction effect of A × B devided into four actions, A1 (level 1) × B1 (level 1), A1 (level 1) × B2 (level 2), A2 (level 2) × B1 (level 1) and A2 (level 2) × B2 (level 2); the statistical analyses were performed using one-way ANOVA.

### 2.3 Drug extract preparation

Drugs from each group (XFZYD 78.0 g; FD disassembled groups: A + B + C + D + E 28.5 g, A + B 10.5 g, A + C + D 18.0 g, A + E 12.0 g, B + C + E 13.5 g, B + D 13.5 g, C 3.0 g, D + E 15.0 g; GUF, 6.0 g, single factor E) were weighed, soaked in water for 1 hour, boiled for 40 min, and extracted repeatedly with deionised water. The combined filtrate was centrifuged at 4°C and 4200r/min for 2 h. After removing the sediment, the filtrate was vacuum-filtered through a 0.45 μm filter and adjusted to a final volume of 100 mL for further experiments. Extracts from different FD disassembled groups were mixed with regular food to obtain specific final concentrations (Supplementary Table S2). The optimal concentration of XFZYD was prepared at 12.5 mg/mL (Wang S. et al., 2020). For GUF, with its concentration in FD as the center (2.0 mg/mL) (Ye, 2022), adjusted by factors of 0.25, 0.5, 1, 2, and 4, resulting in final concentrations of 0.5, 1.0, 2.0, 4.0, and 8.0 mg/mL. All solutions were prepared using deionised water as the standard solvent.

### 2.4 *Drosophila* strain and genetics

*Drosophila* strains and genetic crosses were maintained on cornmeal-agar medium at 25°C in a 12 h light/dark cycle, following standard protocols unless otherwise indicated. The fly strains used are as follows: *w* ^*1118*^ (#3605) and *UAS-*eRFP (#32219) from the Bloomington *Drosophila* Stock Center; *UAS-scrib-IR* (#27424) from the Vienna *Drosophila* RNAi Centre; *ptc*-GAL4 *UAS*-GFP (*ptc>*GFP) and *STAT-*GFP from the Core Facility of *Drosophila* Resource and Technology; and *TRE*-RFP, kindly donated by Professor Xianjue Ma of Westlake University.

Healthy, unmated female flies were randomly grouped for hybridisation. *ptc>*GFP females were crossed with *UAS-scrib-IR* males raised on the standard medium, and their progeny, along with third-instar larvae with the genotype *ptc*>GFP/*UAS-scrib-IR* constituted the model group. Offspring of *ptc*>GFP/*UAS-scrib-IR* larvae raised in a drug-containing medium formed the drug-treated groups. *ptc>*GFP females were crossed with *w* ^*1118*^ males and maintained in a normal medium to collect third-instar larvae with the genotype *ptc*>GFP/+ as the control group. The larvae were raised at 25°C for 48 h and then transferred to 29°C for further feeding as previously described (Wang S. et al., 2020).

### 2.5 High-performance liquid chromatography analysis

Standard chemicals (glycyrrhizic acid (#G111375), liquiritin (#L886004), liquiritigenin (#L115716), and glycyrrhetinic acid (#L110195)) were purchased from Aladdin and Macklin. After grinding, 0.5 g of GUF powder was extracted with 20 mL of methanol via ultrasonic extraction for 2 h. The extracts were analysed using Thermo HPLC U3000 with a Syncronis C18 column (250 mm × 4.6 mm, 5 μm). A 20 μL sample was injected at 30°C, and a 1 mL/min flow rate and detected at 254 nm. The mobile phases were liquiritin and liquiritigenin-methanol (I) and 0.2% phosphoric acid (II) (I: II = 50:50); glycyrrhizic acid-acetonitrile (III) and 0.2% phosphoric acid (IV) (III: IV = 60:40); glycyrrhetinic acid-acetonitrile (V) and 0.05 mol/L potassium dihydrogen phosphate (VI) (V: VI = 60:40). Repeated experiments were conducted to validate precision, accuracy, and linearity of four compounds. Subsequently, the established analytical methods were applied to quantify the four active components in two batches of GUF aqueous extracts.

### 2.6 Food intake

The 3rd instar larvae in all groups were starved for 2 h, fed with a diet containing phosphate-buffered saline (PBS) and 0.8% agar, followed by food supplemented with 0.05% Brilliant Blue FCF (Blue-9) for 20 min. After drying, the larval sample was cleaned with PBS and ground in 100 μL lysate (PBS + 0.1% Triton X-100), then subjected to a previously described protocol (Cao et al., 2022).

### 2.7 ROS staining

The larval samples were incubated in the dark with dihydroethidine (DHE, Beyotime, #S0063), then washed with PBS and fixed in 4% polyformaldehyde (Beyotime, #P0099). Nuclei were stained at room temperature with Hoechst-33342 (Beyotime, #C1025). Finally, samples were dissected and mounted in glycerine (China National Pharmaceutical Group Chemical Reagent Co., Ltd., #10010618).

### 2.8 Immunohistochemistry

The larval samples were fixed in 4% formaldehyde for 20 min, washed with 0.3% phosphate-buffered saline tween-20, incubated overnight with primary antibody at 4°C, and stained with secondary antibody for 4 hours at 25°C, protected from light. Antibodies used: rat anti-E-cadherin (1:100, Developmental Studies Hybridoma Bank (DSHB), #DCAD2), mouse anti-MMP1 (1:200, DSHB, #3A6B4, #14A3D2, #5H7B11), mouse anti-β-integrin (1:100, DSHB, #CF.6G11), goat anti-rat-Cyanine3 (1:1000, Life Technologies, #A10522), goat anti-mouse-Cyanine3 (1:1000, Life Technologies, #A10521). Anti-fluorescence quenched solution with 4′,6-diamino-2-phenylindole (Beyotime, #P0131) was used for mounting. Fluorophores were visualised using an inverted fluorescence system (IX51; Olympus).

### 2.9 Real-time quantitative PCR (RT-qPCR)

RNA was extracted using an RNA extraction kit (Zhongshi Gene, #ZS-M11005) and reverse transcribed using a Supersmart™ cDNA synthesis kit (Zhongshi Gene, #ZS-M14003) following the manufacturer’s instructions. qRT-PCR was performed by real-time PCR (Analytik Jena, qTOWER 2.2) using 2 × SYBR Green premix (Zhongshi Gene, #ZS-M13002). *Rp49* served as the internal control, and relative expressions were calculated using the 2^−ΔΔCt^ method. Primer sequences are listed in Supplementary Table S3.

### 2.10 Data analysis

SPSS 22.0 and GraphPad Prism 9.0 were used for data analysis and graph construction. ImageJ calculated the fluorescence intensity. Orthogonal analysis, one-way ANOVA, Kruskal–Wallis test with Bonferroni correction, or unpaired t-test determined statistical significance. Significance has been denoted as follows: *P* < 0.05: ^*^, ^Δ^ or ^#^; *P* < 0.01: ^**^, ^ΔΔ^ or ^##^; *P* < 0.001: ^***^, ^ΔΔΔ^ or ^###^; *P* < 0.0001: ^****^, ^ΔΔΔΔ^ or ^####^; *P* ≥ 0.05: ns denotes not significant. *P* < 0.05 represents a statistically significant difference. Error bars represent standard deviation. All experiments were repeated at least three times.

## 3 Results

### 3.1 Disassembled recipes of FD

**FIGURE 2:**
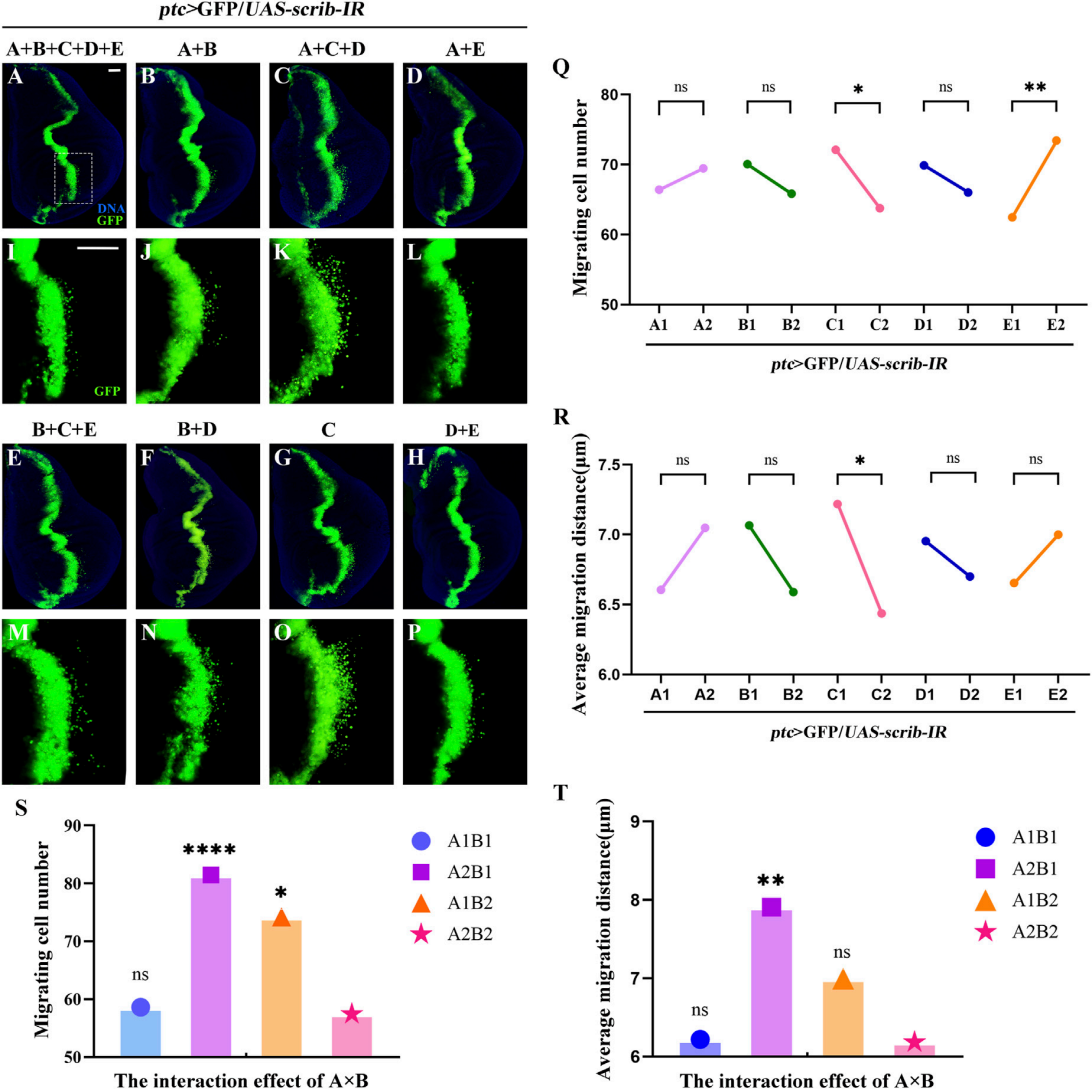
Disassembled recipes of FD inhibit *ptc*>*scirb-IR* induced cell migration. **(A–P)** Fluorescence images representing *ptc*>*scrib-IR* cell migration treated with the disassembled FD recipes, termed A + B + C + D + E, A + B, A + C + D, A + E, B + C + E, C and D + E. I-P are high magnification views of the white dotted boxed areas in a-h, respectively. DAPI (blue) labels the nuclei (DNA); scale bar: 50 µm **(A–P)**. The effect of the five natural herbs on the number of migrating cells [**(Q)**, n = 40] and the mean migration distance [**(R)**, n = 40] is shown. In q and r, the columns from left to right represent the use (1: level 1) and no use (2: level 2) of such drugs. If the mean value at level 1 is lower than that at level 2, and the statistical analysis yielded *P* < 0.05, the use of the drug inhibited cell migration. *P* values were calculated by orthogonal analysis. Data for orthogonal analysis are expressed as means. **(S,T)** The interaction effect of A × B; A1 × B1: A1 (level 1) × B1 (level 1), A1 × B2: A1 (level 1) × B2 (level 2), A2 × B1: A2 (level 2) × B1 (level 1), A2 × B2: A2 (level 2) × B2 (level 2); *P* values were calculated by one-way ANOVA. FD: Five drugs.

To explore key FD components inhibiting cell migration, we designed experimental groups using an orthogonal approach (Table 1). The five herbs, corresponding to factors A, B, C, D, and E, were tested using the *ptc>scrib-IR* cell migration model (Supplementary Figure S1) to assess the effects of these disassembled formulations. GFP-labelled wing disc cells migrated backwards from the anterior/posterior (A/P) axis in all groups (Figures 2A–P). The mean GUF level 1 (E1) was significantly lower than level 2 (E2) (Figure 2Q; *P* < 0.01), indicating that GUF reduces cell migration. Conversely, the mean *Bupleurum* level 1 (C1) was significantly higher than level 2 (C2) (Figures 2Q,R; *P* < 0.05), indicating it promotes migration. The mean migration distance of C1 was also significantly higher than C2 (Figure 2R), implying that *Bupleurum* increases the cell migration distance (Figure 2R). Furthermore, we analysed the interaction effect of A × B. Both A1 (level 1) × B1 (level 1) and A2 (level 2) × B2 (level 2) significantly inhibited the number and distance of cell migration (Figures 2S,T; *P* > 0.05), with no significant difference between them (*P* < 0.05), indicating that either simultaneous use or non-use of these two herbs yields a similar inhibitory effect. In contrast, using either herb alone resulted in a significant promotion of cell migration (Figures 2S,T; *P* < 0.05). Based on the principle of preferring the simplest treatment with the same therapeutic effect, we excluded both herbs from subsequent analyses. Thus, GUF may inhibit cell migration, while *Bupleurum* may have the opposite effect. The other FD herbs (A, B, and D) were found to be ineffective. Therefore, GUF was selected for further exploration of its optimal dosage and underlying mechanisms.

### 3.2 GUF and GUF extract quality analysis

**FIGURE 3:**
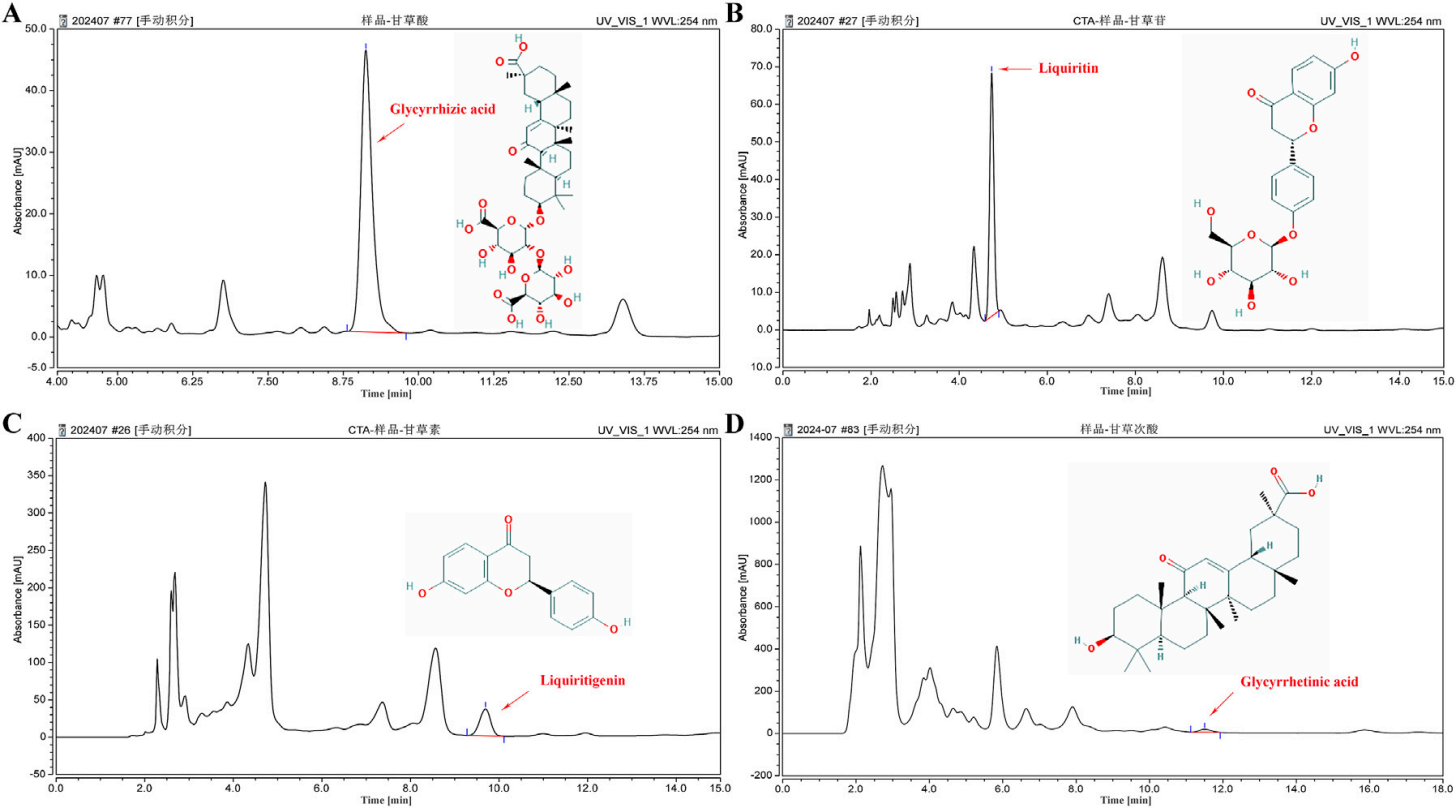
HPLC chromatogram of GUF. HPLC analysis of the four main compounds in GUF sample: glycyrrhizic acid [254 nm, **(A)**], liquiritin [254 nm, **(B)**], liquiritigenin [254 nm, **(C)**], and glycyrrhetinic acid [254 nm, **(D)**]. The red arrows indicate the corresponding peaks. GUF, *Glycyrrhiza uralensis* Fisch.

**FIGURE 4:**
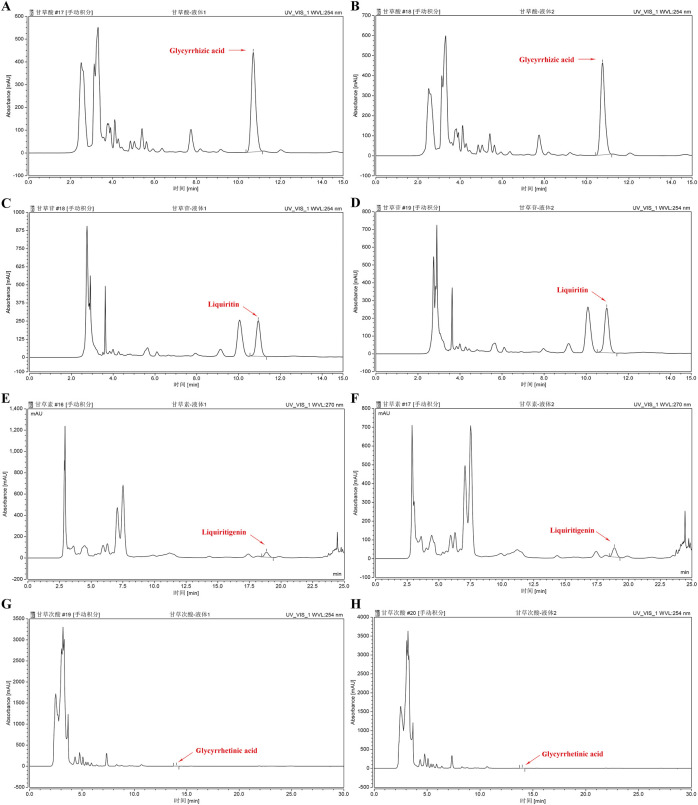
HPLC chromatograms of GUF aqueous extracts from two batches. HPLC analysis of *Glycyrrhiza* aqueous extract sample (batch 1 and 2): glycyrrhizic acid (254 nm, **(A,B)**, liquiritin (254 nm, **(C,D)**, liquiritigenin (270 nm, **(E,F)** and glycyrrhetinic acid (254 nm, **(G,H)**. The red arrows indicate the corresponding peaks. GUF: *Glycyrrhiza uralensis* Fisch.

We assessed the GUF quality using HPLC. Repeated precision evaluations demonstrated relative standard deviations (RSD) below 5% for glycyrrhizic acid (0.2170%), liquiritigenin (0.1473%), liquiritin (0.03557%), and glycyrrhetinic acid (0.1795%). All compounds exhibited excellent linearity (R^2^ ≥ 0.999; Supplementary Figure S3) and accuracy with recoveries ranging from 96.03% to 99.75% (between 95% and 105%). These results confirm the stability of the instrumentation and precision of the analytical methodology. Compared with the standard chromatogram (Supplementary Figure S2), we observed distinct peaks for glycyrrhizic acid (4.669%), liquiritin (1.093%), liquiritigenin (0.09%), and glycyrrhetinic acid (0.065%) in the GUF chemical fingerprints (Figure 3; Table 2). Further comparative analysis revealed consistent peak patterns and comparable contents of active constituents in two batches of GUF aqueous extract (Figure 4; Supplementary Table S4). These results demonstrated the presence of essential active ingredients with stable composition in the samples, making it suitable for further study.

**TABLE 2:** HPLC analysis of GUF representative components.

Sample Name	Height (mAU)	Area (mAU*min)	Sample Size (ug/g)	Proportion (%)
Glycyrrhizic acid	45.759	10.417	46,689.205	4.669
Liquiritin	64.765	7.266	10,928.705	1.093
Liquiritigenin	35.929	10.316	902.2205	0.090
Glycyrrhetinic acid	14.337	5.247	649.294	0.065

GUF: *glycyrrhiza uralensis* fisch.

### 3.3 GUF inhibits *ptc*>*scrib-IR* induced cell migration

**FIGURE 5:**
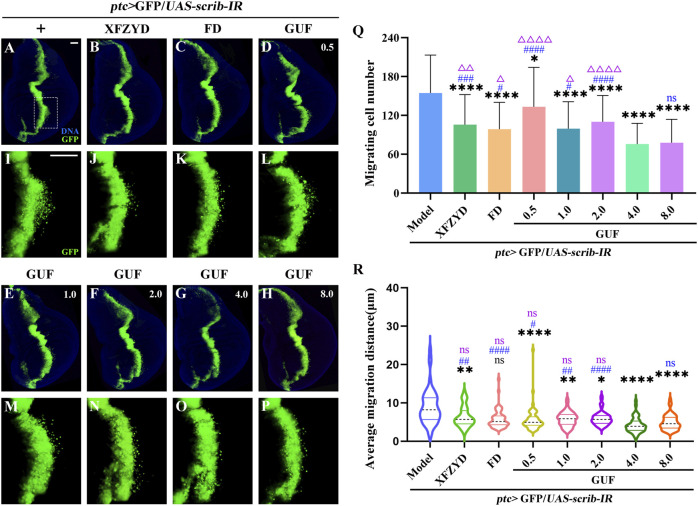
GUF suppresses cell migration. **(A–P)** Representative fluorescent images showing the third instar larva wing discs. The groups include *ptc*>*scrib-IR* without drug treatment (+) and the *ptc*>*scrib-IR* cell migration model treated with XFZYD (12.5 mg/mL), FD (10.0 mg/mL), and GUF at different concentrations (0.5 mg/mL, 1.0 mg/mL, 2.0 mg/mL, 4.0 mg/mL, 8.0 mg/mL). **(I–P)** are high magnification views of the white dotted boxed areas in a-h, respectively. Scale bar: 50 µm **(A–P)**. **(Q)** Statistical chart of cell migration quantity (n = 50) using one-way ANOVA. **(R)** Statistical graph of mean migration distance (n = 50) using the Kruskal-Wallis, with data expressed as quartiles. The Bonferroni multiple comparison test was used for all statistical analyses. Black * and ns represent the comparison between the drug intervention group and the *ptc*>*scrib-IR* without drug treatment group. Blue # and ns represent comparisons between other drug intervention groups and the 4.0 mg/mL GUF group. Purple Δ and ns represent the comparison between the other drug intervention group and the 8.0 mg/mL GUF group. GUF: *Glycyrrhiza uralensis* Fisch.; FD: Five drugs.

To explore the optimal GUF dose for inhibiting cell migration, we prepared media including XFZYD (12.5 mg/mL), FD (10.0 mg/mL) and GUF (0.5–8.0 mg/mL) to feed *ptc*>*scrib-IR* flies from egg to third-instar larval stage. In the model group, GFP-labelled cells in the wing pouch migrated backwards (Figures 5A,I), while all seven drug intervention groups inhibited migration to varying degrees (Figures 5B–H,J–P). Statistical analysis showed that all drug groups inhibited cell migration compared to the model group (Figure 5Q), with GUF (4.0 mg/mL and 8.0 mg/mL) showing the strongest effect (Figure 5Q). No significant difference in inhibition was found between the 4.0 mg/mL and 8.0 mg/mL GUF groups (Figure 5Q). Following the drug simplification principle for equivalent effect, 4.0 mg/mL GUF was the optimal dose for inhibiting migration. All drug groups, except for FD (Figures 5C,K,R), showed inhibitory effects on mean migration distance compared to the model group, with 4.0 mg/mL GUF displaying a distinct effect (Figure 5R). Based on these findings, we concluded that 4.0 mg/mL GUF may be the preferred dose for inhibiting *ptc*>*scrib-IR*-induced cell migration, exceeding the efficacy of XFZYD and FD.

### 3.4 GUF reduces the level of ROS

**FIGURE 6:**
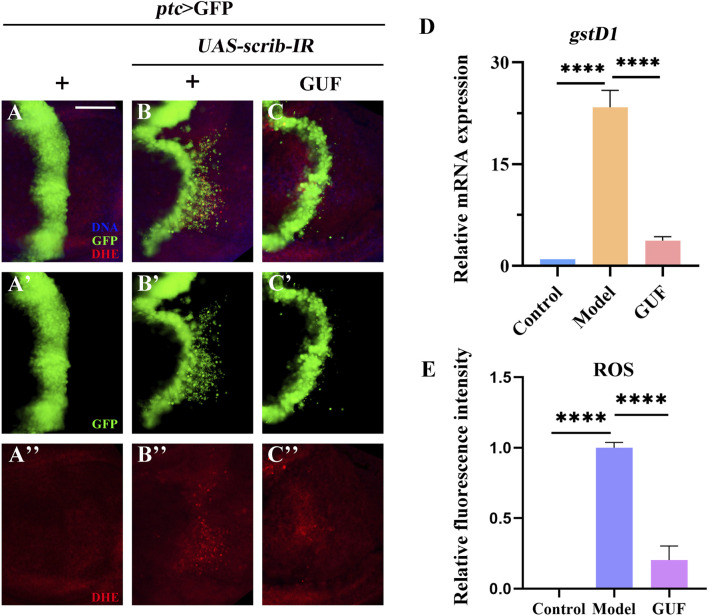
GUF downregulates the level of ROS. **(A–C)** Merged fluorescent images showing the third instar larval wing discs stained with DHE. DAPI (blue) labels the nuclei (DNA); scale bar: 50 µm. A single channel detects only GFP [green, **(A’–C’)**] and DHE [red, **(A”–C”)**]. **(D)** mRNA level of *gstD1* was measured by RT-qPCR assay (n = 3); one-way ANOVA was used to analyse the data. Bonferroni’s multiple comparison tests were used to statistic the *P* value (****P < 0.0001). **(E)** ImageJ evaluated the relative fluorescence intensity of ROS (n = 9–10); one-way ANOVA analysed the data; Bonferroni’s multiple comparison tests calculate the *P* value (****P < 0.0001). GUF: *Glycyrrhiza uralensis* Fisch.; ROS: Reactive oxygen species; DHE: Dihydroethidine; GFP: Green fluorescence protein.

Given the high ROS levels in metastatic tissues (van der Waals et al., 2018), we examined their levels in *Drosophila* under a cell migration background. Compared to the control group (Figures 6A–A”, E), ROS levels increased in the wing pouch of the *ptc*>*scrib-IR* cell migration model (Figures 6B–B”, E). However, GUF at 4.0 mg/mL significantly reduced these levels (Figures 6C–C”, E). Additionally, RT-qPCR showed that mRNA levels of *glutathione s-transferase D1* (*gstD1*), an ROS-promoting factor, were significantly higher in the model group than in the control and were significantly rescued by GUF intervention (Figure 6D). Thus, GUF (4.0 mg/mL) may suppress ROS levels and inhibit cell migration.

**FIGURE 7:**
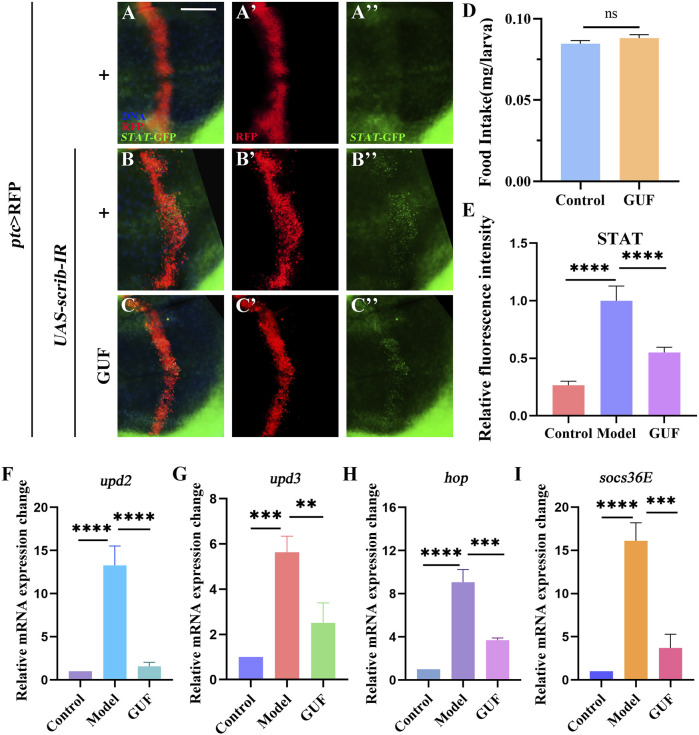
GUF inhibits JAK/STAT signalling activity. **(A–C**) Merged fluorescent micrographs showing the third instar larva wing discs labelled with *STAT*-GFP. DAPI (blue) labels the nuclei (DNA); scale bar: 50 μm. Individual channels detect only RFP [red, **(A’–C’)**] and GFP **(A”–C”)**. **(D)** Food intake measurements for the designated group (n = 3), an unpaired t-test was used to calculate the *P* value. **(E)** ImageJ evaluated the relative fluorescence intensity of *STAT*-GFP (n = 9–10); one-way ANOVA analyzed the data; Bonferroni’s multiple comparison tests calculate the *P* value. **(F–I)** mRNA levels of *upd2*, *upd3*, *hop* and *socs36E* measured by RT-qPCR (n = 3); one-way ANOVA was used to analyse the data. Bonferroni’s multiple comparison tests were employed to calculate the *P* value, with significance levels indicated as ****P < 0.0001, ***P < 0.001, **P < 0.01. GUF: *Glycyrrhiza uralensis* Fisch.; GFP: Green fluorescence protein.

Given that diet may affect cell metabolism and migration, we examined the effect of GUF (4.0 mg/mL) on food intake in flies. Results showed no significant difference in the intake of normal vs GUF-supplemented media (Figure 7D). Thus, we excluded the possibility that feeding rate changes could interfere with cell migration, allowing GUF usage in subsequent experiments.

### 3.5 GUF suppresses JAK/STAT signalling activity

Since the 1990s, the JAK/STAT signalling pathway has been associated with malignancy (Leonard and O'Shea, 1998). To monitor JAK/STAT activity, we overexpressed the *STAT*-GFP (the regulatory region of endogenous STAT fused with GFP) protein *in* the larvae. Compared to the control (Figures 7A–A”, E), *STAT*-GFP expression significantly increased along the A/P axis in *Drosophila* wing discs marked red fluorescent protein (RFP) under the *ptc*>*scrib-IR* background (Figures 7B–B”, E). The upregulated *STAT*-GFP expression was partially rescued by GUF (4.0 mg/mL) (Figures 7C–C”, E). Using RT-qPCR, we found a significant decrease in the mRNA levels of key JAK/STAT factors (*upd2*, *upd3*, *hop*, and *socs36E*) in *ptc>scrib-IR* larvae compared to the controls following GUF treatment (Figures 7F–I)*.* These results indicate that GUF may inhibit *ptc>scrib-IR*-induced cell migration by downregulating JAK/STAT signalling activity.

### 3.6 GUF alters the expression of E-cadherin, MMP1 and β-integrin

**FIGURE 8:**
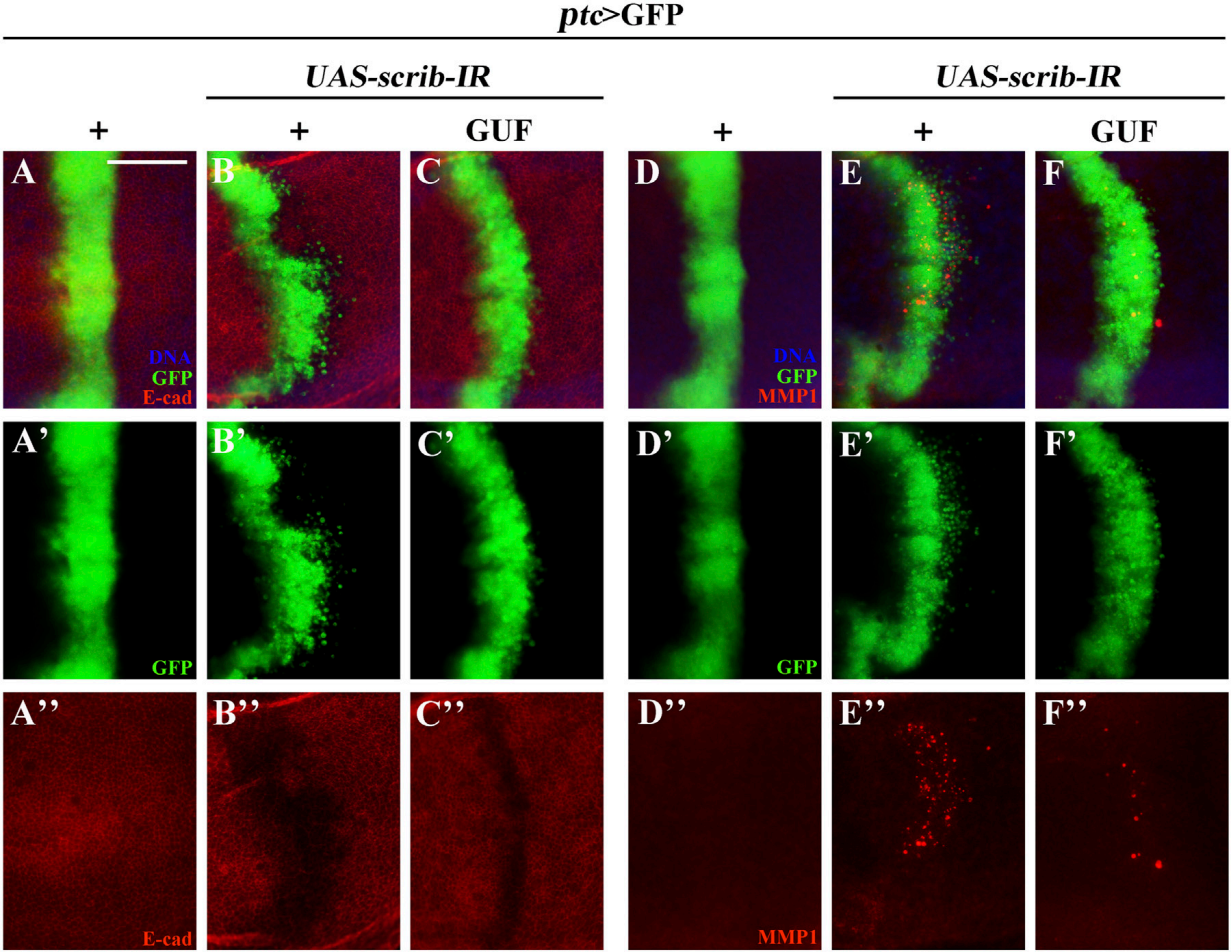
GUF alters E-cadherin and MMP1 expression. Merged fluorescent images showing the third instar larva wing discs stained with anti-E-cadherin (E-cad) **(A–C)** and anti-MMP1 antibodies **(D–F)**. Individual channels detect only GFP [green, **(A’–F’)**], E-cad [red, **(A”–C”)**], and MMP1 [red, **(D”–F”)**]. DAPI (blue) labels the nuclei (DNA); scale bar: 50 μm. GFP, Green fluorescence protein; MMP1, metalloproteinase 1.

EMT is critical for malignant tumour transformation and cancer metastasis (Plygawko et al., 2020). We examined EMT-related proteins (E-cadherin, MMP1 and β-integrin) (Hazan et al., 1997; Maschler et al., 2005; Zhang et al., 2018). Downregulation of the tumour suppressor gene *scrib* along the A/P boundary induced an EMT-like phenotype in *Drosophila* wing discs, by decreasing E-cadherin levels (Figures 8A–A”, B–B”) and increasing MMP1 and β-integrin levels (Figures 8D–D”, E–E”; Supplementary Figures S4A–A”, B–B”). GUF (4.0 mg/mL) significantly restored E-cadherin expression (Figure 8C”) and inhibited MMP1 and β-integrin expression (Figures 8F–F”; Supplementary Figures S4C–C”), suggesting that GUF inhibits cell migration by altering EMT-related factors.

## 4 Discussion

Cancer metastasis is the leading cause of death in patients, yet effective treatments remain limited (Steeg, 2016). Employing the *Drosophila ptc*˃*scrib-IR* cell migration model, we explored the effects of FD on cell migration and the underlying mechanism. GUF, a key inhibitor, showed the strongest effect at 4.0 mg/mL and 8.0 mg/mL. The dose of 4.0 mg/mL for *Drosophila* corresponds to 8.33 g/day for adult human (Deshpande et al., 2014; Slack et al., 2015), aligning with the Chinese Pharmacopoeia guidelines for GUF (2–10 g/day). However, the intake of 8.0 mg/mL is equivalent to the dose of 16.66 g/day for human, which significantly exceeds the upper limit of medication. A meta-analysis and systematic review on the dose and toxicity of *Glycyrrhiza*, suggesting a positive correlation between the daily dose of *Glycyrrhiza* and blood pressure (Penninkilampi et al., 2017). Another study also found that long-term excessive intake of *Glycyrrhiza* can induce hypermineralocorticoidism (Caré et al., 2023). Considering the potential toxicity risk, we selected 4.0 mg/mL as the optimal concentration to carry out the subsequent research. As a classic model organism, *D. melanogaster* is high conserved in metabolism and bioavailability with human (Seidel et al., 2021). It can be used to predict the efficacy of drugs or foods in humans through their effects in *Drosophila*. While it should be noted that considering the multiple factors influencing the effect of drugs in different animal species, like the chemical properties of the drugs, metabolic pathways, and excretion modes, this dosage (8.33 g/day) is only intended as a reference and cannot accurately reflect the actual optimal dosage of GUF for humans.

In an orthogonal design assay, GUF primarily inhibited FD by reducing the number of migrated cells. However, in subsequent testing for the optimal dose, GUF inhibited both the number and distance of migrated cells. This discrepancy may be due to the combined use of other drugs in the orthogonal experiment, where their active ingredients affected the anti-cell migration function, showing no effect on cell migration distance. Further investigation is needed to clarify these interactions. Additionally, we found that *Bupleurum* promotes cell migration. However, previous studies have suggested that *Bupleurum* contains riterpene saponins, volatile oil, flavonoid, lignans, polysaccharide, *etc*, which could play key roles in anti-inflammatory, immune regulation, antioxidant and anti-cancer (Teng et al., 2023). For instance, saikosaponin D (SSD), an important *Bupleurum* component, inhibits colorectal cancer cell proliferation and metastasis by inducing autophagy and apoptosis (Lee et al., 2024). Besides, SSD reduces H_2_O_2_-induced apoptosis of PC12 cells by reducing ROS production and inhibiting MAPK-mediated oxidative damage (Lin et al., 2016). While another research found that SSD can induce the production of ROS and promote oxidative stress responses (Wang et al., 2010). The opposite effects of *Bupleurum* may be related to cell types and drug dosages. In addition, different combinations of natural herbal medicines may affect drug efficacy from another aspect. Although there are currently no studies on *Bupleurum* in combination with other drugs promoting tumor metastasis, all these need further exploration. Clarifying the role of *Bupleurum* in promoting cell migration may help decrease the risk of late-stage metastasis in the treatment of human malignant tumors.

As inflammatory mediators, ROS promote cancer progression by inducing immune escape and stimulating pro-inflammatory responses (Weinberg et al., 2015; Liu et al., 2023). The oxidation-promoting factor, gstD1, positively correlates with ROS levels (Beaver et al., 2012). JAK/STAT signalling mechanisms are highly conserved between *Drosophila* and human, sharing many regulatory factors (Upd2, Upd3, Hop) (Trivedi and Starz-Gaiano, 2018). In *Drosophila*, The increase of ROS further activates upstream cytokines (Upd2 and Upd3) (Chakrabarti and Visweswariah, 2020). Upd and Domeless (Dome) bind extracellularly, activating the JAK/STAT pathway, inducing the phosphorylation of Hopscotch (Hop) (Myllymäki and Rämet, 2014), further phosphorylating *Drosophila* STAT (STAT92E), and transferring it into the nucleus, where STAT92E induces the expression of Socs36E (Kiu and Nicholson, 2012). Socs36E is a *Drosophila* homologue of mammalian cytokine signalling inhibitors (Socs) that negatively regulate JAK/STAT signalling (Callus and Mathey-Prevot, 2002). Thus, we examined the mRNA levels of *gstD1*, *upd2*, *upd3*, *hop*, and *socs36E*; along with ROS and STAT expression *in vivo* to study the ROS-mediated JAK/STAT signalling pathway and found that GUF’s inhibition of cell migration was related to ROS-mediated JAK/STAT activity.

The JAK/STAT signalling pathway regulates immunity, inflammation, tumour development, and other processes (Owen et al., 2019; Sarapultsev et al., 2023). Previous studies demonstrated that multiple compounds, such as aloin and forsythin can inhibit the ROS/JAK/STAT pathway (Ma et al., 2018; Pan et al., 2014). While their inhibitory effects are primarily attributed to anti-inflammatory properties, evidence supporting their anti-tumor efficacy remains limited. In contrast to single compound, such as aloin or forsythin, *Glycyrrhiza* contains over 400 compounds with multiple functions, including anti-inflammatory, antioxidant, anti-tumor, and immunomodulatory activities. Among these, glycyrrhizic acid, liquiritin, liquiritigenin, glycyrrhetinic acid, and their derivatives are present in relatively large concentrations and are recognized for their biological activities (He et al., 2023). Glycyrrhizic acid can inhibit the invasion and migration of human erythroleukemia cells by mediating the AKT/mTOR/STAT3 pathway activity (He et al., 2015). Its salt form, glycyrrhizin, suppresses lung tumor growth in mice by downregulating the JAK/STAT signaling pathway (Wu et al., 2018), and specifically targets the JAK/STAT1 pathway to reduce its activity in human immortalized keratinocyte (HaCaT) cells (Xu et al., 2018). Moreover, glycyrrhizin induces apoptosis in liver cancer cells by acting on the ROS-mediated MAPK/Akt/NF-kB signaling pathway (Wang J. R. et al., 2020). Isoliquiritin downregulates the JAK/STAT signaling pathway by inhibiting the phosphorylation of JAK2, STAT1, STAT3, and STAT5, thereby regulating immune and inflammation responses and contributing to the treatment of atopic dermatitis (Wu et al., 2022). Conversely, isoliquiritigenin as an antioxidant, promotes differentiation of leukemia monocytes, facilitates reversal of tumor cells, and supports recovery of cancer patients (Li et al., 2009). Although each individual compound may exert effects in inflammation, oxidative stress, immunity and tumors, The combined effect of multiple compounds in GUF have been shown to be more effective than the single one (He et al., 2023). Our study explored GUF’s suppressive effect on cell migration through oxidative stress, the JAK/STAT pathway, and EMT in *Drosophila in vivo*. HPLC preliminarily analysed the main active components, providing insights into GUF’s comprehensive effect on tumour metastasis.

Previous studies have shown that inhibiting JAK/STAT signalling can reverse EMT cancer cells, reducing invasion and metastasis (Yang et al., 2021). E-cadherin, β-integrin, and MMP1 are key regulators in the EMT process. E-cadherin functions as a tumor suppressor protein, and its downregulation facilitates EMT and induces tumor metastasis by suppressing GSK3β-mediated β-catenin phosphorylation and upregulating Twist, an EMT transcription factor (Fang and Kang, 2021). The integrins adhere to the extracellular matrix (ECM) surface and regulate cell invasion and metastasis by influencing the localization and activity of matrix-degrading proteases, such as urokinase-type plasminogen activator (uPA) and matrix metalloprotease 2 (MMP2) (Hamidi and Ivaska, 2018). β-integrin regulates cell adhesion and migration, playing a central role in EMT (Maschler et al., 2005). MMP1, a matrix metalloproteinase, promotes cancer progression by degrading the extracellular matrix (Zhang et al., 2018). In head and neck squamous cell carcinoma cells, silencing the MMP1 gene by siRNA significantly inhibits cell proliferation, migration, and invasion, and activates apoptosis through the EMT process (Zhang et al., 2022). Our study found that *Glycyrrhiza* may inhibit the EMT process by modulating MMP1, β-integrin and E-cadherin expression, inhibiting cell migration.

*Drosophila* and human are highly conserved in the core regulatory factors of EMT, such as the Snail family and Twist gene, both of which are involved in the downregulation of E-cadherin, thereby providing a simple model for studying the EMT process and facilitating the analysis of the underlying mechanisms (Bell and Thompson, 2014; Wong et al., 2014). However, due to differences in genetic complexity, EMT signaling pathways in *Drosophila* are comparatively simpler and rely predominantly on the core transcription factors snail and twist (Baum et al., 2008). While in humans, the EMT regulated by multiple factors, such as Snail, Twist, Zeb, and Slug (Huang et al., 2022). Conversely, the EMT process in *Drosophila* is primarily characterized by collective migration, whereas the human cancer cells typically exhibit greater invasiveness (Campbell et al., 2019; Huang et al., 2022). Due to its limited physiological complexity, *Drosophila* only recapitulates the EMT phenomenon, making it challenging to study the intricate regulatory mechanism within the metastatic microenvironment (Pocha and Montell, 2014; Taki et al., 2021). As a result, differences in drug efficacy may arise between *Drosophila* and human. Thus, further validation using human cancer lines (e.g., melanoma, lung cancer, gastric cancer) and mammalian *in vivo* models is warranted to validate the efficacy of GUF, and provide experimental evidence for its clinical application in the prevention and treatment of malignant tumor metastasis across specific disease types.

## 5 Conclusion

In this study, we found that GUF inhibited cell migration by suppressing ROS-mediated JAK/STAT signalling and the EMT process, providing novel insights for the clinical treatment of cancer and other related diseases.

## Data Availability

The original contributions presented in the study are included in the article/Supplementary Material, further inquiries can be directed to the corresponding authors.
