# Supplementary material for: Glycyrrhiza uralensis Fisch. suppresses cell migration via ROS and JAK/STAT signalling pathways in Drosophila
Source: Front Pharmacol. 2025 Jun 27;16:1549920. doi: 10.3389/fphar.2025.1549920 (PMC12246979; doi:10.3389/fphar.2025.1549920)
Supplement: Supplementary file 5 [file DataSheet1.docx]

Supplementary Material

***Glycyrrhiza uralensis* Fisch. Inhibit Cell Migration via ROS and JAK/STAT signaling pathway in *Drosophila***

**Supplementary Table 1. Details of XFZYD (one dose)**

| **Latin name**  **(Chinese name)** | **Batch Number** | **Origin** | **Amount (g)** |
| --- | --- | --- | --- |
| *Prunus persica* (L.) Batsch  (Tao Ren) | 291220701 | Liaoning, China | 12.0 |
| *Carthamus tinctorius* L.  (Hong Hua) | 029220701 | Xinjiang, China | 9.0 |
| *Ligusticum chuanxiong* Hort.  (Chuan Xiong) | 125220101 | Sichuan, China | 4.5 |
| *Angelica sinensis* (Oliv.) Diels.  (Dang Gui) | 132201201 | Gansu, China | 9.0 |
| *Paeonia lactiflora* Pall.  (Chi Shao) | 123211101 | Heilongjiang, China | 6.0 |
| *Rehmannia glutinosa* Libosch.  (Sheng Dihuang) | c22082610 | Henan, China | 9.0 |
| *Bupleurum chinense* DC*.*  (Bei Chaihu) | 119210702 | Shanxi, China | 3.0 |
| *Citrus aurantium* L.  (Fuchao Zhiqiao) | 310210801 | Jiangxi, China | 6.0 |
| *Glycyrrhiza uralensis* Fisch.  (Gan Cao) | 144210401 | Xinjiang, China | 6.0 |
| *Cyathula officinalis* Kuan  (Chuan Niuxi) | 124210501 | Sichuan, China | 9.0 |
| *Platycodon grandiflorum* (Jacq.) A. DC.  (Jie Geng) | 173200701 | Hebei, China | 4.5 |

**Supplementary Table 2. Concentration conversion of disassembled components of FD**

| **Group** | **Concentration Conversion Formula**  **(X/28.5×10.0mg/ml)** | **Final concentration**  **(mg/mL)** |
| --- | --- | --- |
| A+B+C+D+E | 28.5/28.50×10.0 | 10.0 |
| A+B | 10.5/28.50×10.0 | 3.7 |
| A+C+D | 18.0/28.50×10.0 | 6.3 |
| A+E | 12.0/28.50×10.0 | 4.2 |
| B+C+E | 13.5/28.50×10.0 | 4.7 |
| B+D | 13.5/28.50×10.0 | 4.7 |
| C | 3.0/28.50×10.0 | 1.1 |
| D+E | 15.0/28.50×10.0 | 5.3 |

**Supplementary Table 3. Primer sequences**

| Gene | Forward Primer | | Reverse Primer |
| --- | --- | --- | --- |
| *Rp49* | CTTCATCCGCCACCAGTC | | GCACCAGGAACTTCTTGAATC |
| *gstD1* | GACCGACTCCCTGTACCCTAA | | GCGGCCTCGATCTTCTTGA |
| *upd2* | GCGGTGAAGCTAAAGACTTGG | | CATTGCTGTTCGGATAGGAGG |
| *upd3* | | CGGAGCGGTAACAAAACGG | GGAGAGGGCAAACTGGGACAT |
| *hop* | | GTGGGCTCCAAGATACG | GGCAGATACTGAACGGTG |
| *socs36E* | | CGCCCTGTCTGCGGATTC | GGGCTGTCGTTCGGTTTTG |

**Supplementary Table 4. HPLC chromatograms of GUF aqueous extracts from two batches**

| **Peak identification** | **Retention time**  **(min)** | **Peak area**  **(mAU*min)** | **Peak height**  **(mAU)** | **Sample amount**  **(ug/g)** |
| --- | --- | --- | --- | --- |
| Glycyrrhizic acid (Batch 1) | 10.713 | 108.284 | 438.168 | 2117.0224 |
| Glycyrrhizic acid (Batch 2) | 10.750 | 110.873 | 459.573 | 2105.7754 |
| Liquiritin  (Batch 1) | 10.943 | 60.723 | 246.865 | 736.3060 |
| Liquiritin  (Batch 2) | 10.977 | 63.732 | 253.778 | 746.4939 |
| Liquiritigenin  (Batch 1) | 18.848 | 17.283 | 46.388 | 47.517 |
| Liquiritigenin  (Batch 2) | 18.870 | 18.100 | 49.360 | 48.3434 |
| Glycyrrhetinic acid (Batch 1) | 14.020 | 1.050 | 4.581 | 3.4193 |
| Glycyrrhetinic acid (Batch 2) | 14.003 | 1.184 | 5.282 | 3.7449 |

**Supplementary Figure 1. *ptc*>*scrib-IR* promotes massive cell migration.**

Representative fluorescent images showing the third-instar larval wing discs. **A** and **C** are the *ptc*>GFP/+ control, **B** and **D** are the *ptc*>GFP/*UAS-scrib-IR* cell migration model. DAPI (blue) labels the nuclei (DNA); scale bar: 50 µm.

**Supplementary Figure 2. The HPLC chromatogram of standard controls.**

HPLC analysis of the standards: glycyrrhizic acid (254nm, **A**), liquiritin (254nm, **B**), liquiritigenin (254nm, **C**) and glycyrrhetinic acid (254nm, **D**). The red arrows indicate the corresponding peaks.

**Supplementary Figure 3. The linear analysis and standard curve of HPLC.**

The linear analysis and standard curve of the four compounds: glycyrrhizic acid, liquiritin, liquiritigenin, and glycyrrhetinic acid.

GUF: *Glycyrrhiza uralensis* Fisch.

**Supplementary Figure 4. GUF suppresses β-integrin expression.**

Merged fluorescent images showing the third-instar larval wing discs stained with anti-β-integrin antibody (**A-C**). Individual channels detect only GFP (green, **A’**-**C’**) and β-integrin (red, **A”**-**C”**). DAPI (blue) labels the nuclei (DNA); scale bar: 50 μm.

GUF: *Glycyrrhiza uralensis* Fisch.; GFP: Green fluorescence protein.
